# BMC Women’s Health reviewer acknowledgement 2014

**DOI:** 10.1186/s12905-015-0168-3

**Published:** 2015-03-15

**Authors:** Nawsheen Boodhun

**Affiliations:** BioMed Central, Floor 6, 236 Gray’s Inn Road, London, WC1X 8HB UK

## Abstract

The editors of BMC Women’s Health would like to thank all our reviewers who have contributed to the journal in Volume 14 (2014).

**Omalkhair Abulkhair**

Saudi Arabia

**Richard Adanu**

Ghana

**Abiodun Adewuya**

Nigeria

**Amha Admasie**

Ethiopia

**Leonard Ajah**

Nigeria

**Angela Akol**

Uganda

**Tit Albreht**

Slovenia

**Sami Al-Dubai**

Malaysia

**Mussie Alemayehu**

Ethiopia

**Tazeen Saeed Ali**

Pakistan

**Martin Almquist**

Sweden

**Redhwan Al-Naggar**

Malaysia

**Nisreen Alwan**

UK

**Isabel Amendoeira**

Portugal

**Amanda Amos**

UK

**Amanda Ampt**

Australia

**Eleanor Anderson**

USA

**Rosita Aniuliene**

Lithuania

**Augustine Ankomah**

Ghana

**Edward Araujo Júnior**

Brazil

**Puneet Arora**

India

**Ryoko Asano**

Japan

**Esther Asekun-Olarinmoye**

Nigeria

**Smita Asthana**

India

**Erika Austin**

USA

**Maria Marcedes Avila**

Argentina

**Hamdy Azim**

Egypt

**Bontha Babu**

India

**Valerio Bacak**

USA

**Sabrina Bakeera-Kitaka**

Uganda

**Frank Bally**

Switzerland

**Maggi Banning**

UK

**Kouji Banno**

Japan

**Lesley Barclay**

Australia

**Janine Barden-O'Fallon**

USA

**Jane Barlow**

UK

**Pedro N Barri**

Spain

**Raymond E Baser**

USA

**Karine Baumstarck**

France

**Annette Beautrais**

USA

**Mebratu Bekele**

Ethiopia

**Ruud Bekkers**

Netherlands

**Tefera Belachew**

Ethiopia

**Suzanne Belton**

Australia

**Ian Bennett**

Australia

**Marge Berer**

UK

**Wendie Berg**

USA

**Yemane Berhane**

Ethiopia

**Sveinung Berntsen**

Norway

**Haimanti Bhattacharya**

USA

**Junaid Bhatti**

France

**Colin Binns**

Australia

**Charlotte Björkenstam**

Sweden

**Susanne Blödt**

Germany

**Barbara Body**

USA

**Fiona Boland**

Ireland

**Stefan Bösner**

Germany

**Alex Bottle**

UK

**Jacqueline Boyle**

Australia

**Hillary Bracken**

USA

**Kathryn Branch**

USA

**Lynn Breau**

Canada

**Mette Brekke**

Norway

**Hans Brölmann**

Netherlands

**Gill Brook**

UK

**Nele Brusselaers**

Sweden

**Anna Brytek-Matera**

Poland

**Lauro Bucchi**

Italy

**Thanh Bui**

Viet Nam

**Marrije Buist**

Netherlands

**Margit Burmeister**

USA

**Igor Burstyn**

USA

**Hale Basak Caglar**

Turkey

**Angelo Cagnacci**

Italy

**Jean Calleja Agius**

Malta

**Sharon Cameron**

UK

**Giampiero Capobianco**

Italy

**Francisco Carmona**

Spain

**Nicholas Carris**

USA

**Bethany Caruso**

USA

**Luis Carvajal Carmona**

USA

**Luis Carvajal Carmona**

UK

**Tanya Caulfield**

Australia

**Gisella Cavallo**

Italy

**Tyrone Ceaser**

USA

**Venkatesan Chakrapani**

India

**Eric Chamot**

USA

**Sun Ju Chang**

Korea, South

**Jane Chiu**

Taiwan

**Catherine Chojenta**

Australia

**Cari Clark**

United States Minor Outlying Islands

**Melissa Clark**

USA

**John Cleland**

UK

**Barbara Cochrane**

USA

**Ruth Coleman**

UK

**Helen Conaglen**

New Zealand

**Kerith Conron**

USA

**Dominic Conroy**

UK

**Lorraine Corfield**

UK

**Denise Cote-Arsenault**

USA

**Jane Cover**

USA

**Frances Cowan**

UK

**Catherine Crespi**

USA

**Sudhamshu Dahal**

Nepal

**Koustuv Dalal**

Sweden

**Anne Kjersti Daltveit**

Norway

**Dionne Dames-Rahming**

Bahamas

**Anindita Dasgupta**

USA

**Kim De Jong**

Netherlands

**Peter De Jong**

South Africa

**Hélène Delisle**

Canada

**Boris Denisov**

Russian Federation

**Teresa Depineres**

Colombia

**Haryana Dhillon**

Australia

**Cyril Dim**

Nigeria

**Cyril Dim**

Norway

**Guzel Discigil**

Turkey

**Bosiljka Djikanovic**

Serbia

**Mai Do**

USA

**David Teye Doku**

Ghana

**Pat Doyle**

UK

**Silke Dyer**

South Africa

**Suzanne Dyson**

Australia

**Joanna Dytfeld**

Poland

**Ilana Dzuba**

USA

**Katie Edwards**

USA

**István Egerszegi**

Hungary

**Murat Ekin**

Turkey

**Samar El Khoudary**

USA

**Yeetey Enuameh**

Ghana

**Karen Ertel**

USA

**Cam Escoffery**

USA

**Ellen Evans**

USA

**Jemma Evans**

Australia

**Amy Eyler**

USA

**Ifeanyichukwu Ezebialu**

Nigeria

**Chen Fangfang**

China

**Hamdad Farida**

France

**Arnaud Fauconnier**

France

**Olufunmilayo Fawole**

Nigeria

**Asghar Fazaeli**

Iran

**Bradford Fenton**

USA

**Gillian Fletcher**

Australia

**Maralyn Foureur**

Australia

**Betsy Foxman**

USA

**Kathleen Franchek-Roa**

USA

**Jan C. Frich**

Norway

**Alfonso Frigerio**

Italy

**Caren Frost**

USA

**Allen Furr**

USA

**Donald Fylstra**

USA

**C. Ann Gakumo**

USA

**Hadiza Galadanci**

Nigeria

**Rashmi Gangamma**

USA

**Kaniz Gausia**

Australia

**Abebaw Gebeyehu**

Ethiopia

**Seifu Gebreyesus**

Ethiopia

**Sadie Geraghty**

Australia

**Luca Giannella**

Italy

**Peter Gichangi**

Kenya

**Jean Gilmour**

New Zealand

**Mercè Giner**

Spain

**Malin Gingnell**

Sweden

**Livia Giordano**

Italy

**Paolo Giorgi Rossi**

Italy

**Mika Gissler**

Finland

**Norman Goldstuck**

South Africa

**Chaitra Gopalappa**

USA

**Kathleen Gray**

Australia

**Christoph Grimm**

Austria

**Rosalie Grivell**

Australia

**Cynthia Gross**

USA

**Sylvia Guendelman**

USA

**Hediye Pinar Gunbey**

Turkey

**Boliang Guo**

UK

**Nalini Gupta**

India

**Robert Gutman**

USA

**Deepak Gyenwali**

Nepal

**Lisa Haddad**

USA

**Demewoz Haile**

Ethiopia

**Nafisa Halim**

USA

**Amy Hammock**

USA

**Charlotte Hanlon**

Ethiopia

**Laura Harris**

USA

**Angela Hassiotis**

UK

**Ari Haukkala**

Finland

**Kristiann Heesch**

Australia

**Oskari Heikinheimo**

Finland

**Deborah Helitzer**

USA

**Ingrid Hellstrom**

Sweden

**Etienne Henn**

South Africa

**Fernando Hernanz**

Spain

**Helen Herrman**

Australia

**Paul Hewson**

UK

**Lisa Hines**

USA

**Jane Hocking**

Australia

**Eleanor Holroyd**

Australia

**Sara Holton**

Australia

**Michie Hosokawa**

Japan

**Tadayuki Iida**

Japan

**Jennifer D. Irwin**

Canada

**Rafiqul Islam**

Bangladesh

**Hajime Iwasa**

Japan

**Remigius Izunwa**

South Africa

**Pascal Izzicupo**

Italy

**Robert Jackson**

USA

**Bernard Jacquetin**

France

**Chowdhury Jalal**

Canada

**Jos Jayarajan**

Australia

**Stephen Jeffery**

South Africa

**Laura Jones**

USA

**Frank Kaharuza**

Uganda

**László Kalabay**

Hungary

**Ngianga Ii Kandala**

UK

**Ngianga-Bakwin Kandala**

UK

**Rajendra Karkee**

Nepal

**Kristina Karvinen**

Canada

**Getnet Kassie**

Ethiopia

**Ravneet Kaur**

India

**Sarah Keadle**

USA

**Dana Kelly**

USA

**David Kendler**

Canada

**Afsaneh Keramat**

Iran

**Mmh Khan**

Germany

**Nazli Khatib**

India

**Olga Khavjou**

USA

**Tae-Hee Kim**

Korea, South

**Mi-La Kim**

Korea, South

**Sun Kim**

USA

**Bruce Kimler**

USA

**Evan Kleiman**

USA

**M. Tish Knobf**

USA

**Patrick Kolsteren**

Belgium

**Liisa Korkalo**

Finland

**Sara Kornfield**

USA

**Elise Kosunen**

Finland

**Zoltan Kozinszky**

Sweden

**Jayashri Kulkarni**

Australia

**Abhishek Kumar**

India

**Hannah Kuper**

UK

**Richard Kwok**

USA

**Sharon Laing**

USA

**Yihunie L Lakew**

Ethiopia

**Maya Lambiase**

USA

**Pham Thi Lan**

Viet Nam

**Kuo-Chung Lan**

Taiwan

**Rebecca Landy**

UK

**Per-Göran Larsson**

Sweden

**Arthur Leader**

Canada

**Raymond Lee**

Canada

**Guillaume Legendre**

France

**Deborah Legorreta**

Mexico

**Iñaki Lete**

Spain

**Janni Leung**

Australia

**Andrew Levy**

UK

**Wilson Liambila**

Kenya

**Sharon Licqurish**

Australia

**Michal Liebergall**

Israel

**Chih-Ming Lin**

Taiwan

**Alexandre Lobo**

Brazil

**Virginia Lope**

Spain

**Keira Lowther**

UK

**Jean-Philippe Lucot**

France

**Madani Ly**

Mali

**G. William Lyerly**

USA

**Stéphanie Madill**

Canada

**Qun Mai**

Australia

**Kingshuk Majumder**

UK

**Fredrick Makumbi**

Uganda

**Delayehu Bekele Mamo**

Ethiopia

**Ivanny Marchant**

Chile

**Spyros Marinopoulos**

Greece

**Renee Masching**

Canada

**Saba Masho**

USA

**Linda Mason**

UK

**Leslie Massad**

USA

**Paola Mastromarino**

Italy

**Shigeki Matsubara**

Japan

**Roy William Mayega**

Uganda

**Anthony Mbonye**

Uganda

**Arthur Mccausland**

USA

**Ellie Mcdonald**

Australia

**Sue Mckenzie-Mohr**

Canada

**Ivo Meinhold-Heerlein**

Germany

**Marius Meintjes**

USA

**Yohannes Adama Melaku**

Ethiopia

**Zelalem Birhanu Mengesha**

Ethiopia

**Sylvia Metcalfe**

Australia

**Kara Michels**

USA

**Ana Miklavcic Visnjevec**

Slovenia

**Joanna Mishtal**

USA

**Anu Molarius**

Sweden

**Myo-Myo Mon**

Myanmar

**Ali Montazeri**

Iran

**Kathryn Moracco**

USA

**Stephen Morrell**

Australia

**Dieudonne Muhoza**

Rwanda

**Sunni Mumford**

USA

**Marshall Munjoma**

Zimbabwe

**Jenny Munro**

Australia

**Terra Murray**

Canada

**Twaha Mutyaba**

Uganda

**Richard Muwonge**

France

**Ludovico Muzii**

Italy

**Zehra Naqvi**

Pakistan

**Zachary Nash**

UK

**Laura Nellums**

UK

**Patrick Neven**

Belgium

**Bernie Newman**

USA

**Nnabuike Chibuoke Ngene**

South Africa

**Hanh Nguyen**

Viet Nam

**Ioannis Nikolopoulos**

UK

**Nusrat Nisar**

Pakistan

**Lena Nordgren**

Sweden

**Daniel Noyola**

Mexico

**Kenneth Nwankwo**

Nigeria

**Theophilus Nwankwo**

Nigeria

**Francis Obare**

Kenya

**Samuel Obi**

Nigeria

**Milan Obradovic**

Serbia

**Björn Oddens**

Netherlands

**Akin-Tunde Odukogbe**

UK

**Funmilola Olaolorun**

Nigeria

**Abiodun Omole-Ohonsi**

Nigeria

**Ken Ong**

UK

**Samuel Antwi Oppong**

Ghana

**Areej Othman**

Jordan

**Sharon O'Toole**

Ireland

**Santiago Palacios**

Spain

**Maria Papadakaki**

Greece

**Dimitrios Papoutsis**

UK

**Carlos Passos**

Brazil

**Mary Ellen Pavone**

USA

**Michael Pearl**

USA

**Erin Pearson**

USA

**Anna Pease**

UK

**Nasheeta Peer**

South Africa

**Dori Pekmezi**

USA

**Karl Peltzer**

South Africa

**Karl Peltzer**

Thailand

**Tirso Perez-Medina**

Spain

**Patrick Petignat**

Switzerland

**Vorapong Phupong**

Thailand

**Olivier Picone**

France

**Kiran Pienaar**

Australia

**Oriol Porta**

Spain

**Kalpana Poudel-Tandukar**

Japan

**Rakesh Ps**

India

**Sibylle Rahlenbeck**

Germany

**Silvina Ramos**

Argentina

**Sara Randall**

UK

**Shirley Randell Ao**

Australia

**Kimmo Räsänen**

Finland

**Manee Rattanachaiyanont**

Thailand

**Fernando Reis**

Brazil

**Angel Remacha**

Spain

**Otter Renee**

Netherlands

**Heidi Resnick**

USA

**Musarrat Riaz**

Pakistan

**Paolo Ricci**

Chile

**Gunter Rienhardt**

UK

**Cheryl Robbins**

USA

**Beatriz Rojas**

Spain

**Thomas Römer**

Germany

**Robin Room**

Australia

**Andre Rose**

South Africa

**Sue Ross**

Canada

**Ingrid Rowlands**

Australia

**Benjamin Rybicki**

USA

**Sonia Salari**

USA

**Bodour Salhia**

USA

**Ana Paula Sampaio Carvalho**

Portugal

**Ingvild Sandøy**

Norway

**Haribondhu Sarma**

Bangladesh

**Peter Sasieni**

UK

**Stephanie Sassoon**

USA

**Alexandra Sawyer**

UK

**Andreia Schineanu**

Australia

**Yu-Mei Schoenberger**

USA

**Hilary Schwandt**

USA

**Peter Schwartz**

USA

**Marc Schwartz**

USA

**Kate Seear**

Australia

**Nadine Seward**

UK

**Babar Tasneem Shaikh**

Pakistan

**Donat Shamba**

Tanzania

**Abhishek Sharma**

USA

**Nidhi Sharma**

India

**Tarek Shokeir**

Egypt

**Binjwala Shrestha**

Nepal

**Holly Sienkiewicz**

USA

**Leickness Chisamu Simbayi**

South Africa

**Melissa Simon**

USA

**Kuldip Singh**

India

**Nilanchali Singh**

India

**Savita Singhal**

India

**Jolene Skordis-Worrall**

UK

**Lidwien Smit**

Netherlands

**Paige Smith**

USA

**Leon Snyman**

South Africa

**Hirohito Sone**

Japan

**Carlos Souza**

Brazil

**Dawn Stacey**

Canada

**Judith Stephens**

Ghana

**Zoe Stewart**

UK

**Jamila Stockman**

USA

**Annika Strandell**

Sweden

**Saverio Stranges**

UK

**Carol Strong**

Taiwan

**H. Irene Su**

USA

**Carmen Suárez**

Spain

**Minoru Sugiura**

Japan

**Farhana Sultana**

Bangladesh

**Rapeepong Suphanchaimat**

Thailand

**Elisabeth Svensson**

Sweden

**Angela Taft**

Australia

**Sharon Talboys**

USA

**Anthony Mwinilanaa Tampah-Naah**

Ghana

**Amanda Tanner**

USA

**Cetin Tanrikulu**

Turkey

**Yibeltal Tebekaw**

Ethiopia

**Brigitte Tenni**

Australia

**Harish Thippeswamy**

India

**David Thomas**

USA

**Jane Thompson**

Australia

**Gill Thomson**

UK

**Christiana Titaley**

Indonesia

**Catherine Todd**

USA

**Elena Toffol**

Finland

**Gerald Tomkin**

Ireland

**Thach Tran**

Viet Nam

**Lyndal Trevena**

Australia

**Alessandra Trovó De Marqui**

Brazil

**Fuu-Jen Tsai**

Taiwan

**Emmanuel Onyebuchi Ugwu**

Nigeria

**Uchenna Umeh**

Nigeria

**Zephne M. Van Der Spuy**

South Africa

**Hans Van Geelen**

Netherlands

**David Vasquez-Awad**

Colombia

**Freija Verdoodt**

Belgium

**Oriana Vesga**

USA

**Simone Vigod**

Canada

**Paulina Villaseca**

Chile

**Carol Vlassoff**

Canada

**Zeleke Alebachew Wagaw**

Ethiopia

**Denis Walsh**

UK

**Peng-Hui Wang**

Taiwan

**Cuili Wang**

China

**Rhoda Wanyenze**

Uganda

**Narelle Warren**

Australia

**Griffin Weber**

USA

**Nuwan Wickramasinghe**

Sri Lanka

**Lisa Wigfall**

USA

**Joellen Wilbur**

USA

**Dirk Wildemeersch**

Belgium

**Shawna Willey**

Uzbekistan

**Shawna Willey**

USA

**Bianca Wilson**

USA

**Cynthia Woodsong**

South Africa

**Cynthia Woodsong**

USA

**Jeremy Wright**

Ethiopia

**Jie Wu**

China

**Ming-Ping Wu**

Taiwan

**Sara Wuehler**

Ethiopia

**Rebecca Wurtz**

USA

**Jinsong Xiao**

China

**Fan Xu**

China

**Hayat Yalin**

Turkey

**Hakan Yarali**

Turkey

**Jennifer Yarger**

USA

**Kang Youngmi**

Korea, South

**Sofija Zagarins**

USA

**Desalegn Zegeye**

Ethiopia

**Berihun Megabiaw Zeleke**

Ethiopia

**Chen Zhang**

USA

**Rong Zhang**

China

**Qin Zhuo**

China

**Karin Zingmark**

Sweden

**Antonella Zucchetto**

Italy

